# Severe Affection of an Eye, Resulting from a Diseased Tooth

**Published:** 1866-04

**Authors:** Jos. S. Hildreth

**Affiliations:** Late Surgeon in charge of the U.S. Army Eye and Ear Hospital, Chicago


ARTICLE XIV.

### SEVERE AFFECTION OF AN EYE, RESULTING FROM A DISEASED TOOTH.

REPORTED BY JOS. S. HILDRETH, M.D., late Surgeon in charge of the U.S. Army Eye and Ear Hospital, Chicago.

On the 9th of last month, I was called by Dr. Lilly, of this city, to treat Mary ———, age 12 years.

Her condition was as follows:—The globe of the left eye, quite immovable by the recti muscles, was forced upwards. The upper lid covered about five-eighths of the cornea, the lower was forced downward and outward. The inferior conjunctival "cul de sac" was inverted and projecting from the commissure of the lids. The cornea was ulcerating at its lower periphery; but this membrane was not anæsthetized. The pupil had become dilated and fixed. The second superior temporary molar of that side was carious and loose; the gum ulcerating and the cheek swollen. Tenderness existed along the inferior border of the orbit, especially near the foramen, and extended into the canine fossa, where it was more marked. Great pain in and about the orbit, but little from the teeth. Pressure with the finger on the inner and lower margin of the orbit produced pain in the tooth; and pressure on the affected tooth augmented the pain in the orbit. Vision with this eye so affected as not to be able to count the fingers at any distance.

This condition of the patient commenced ten days before, by pain in the tooth. On the third day the eye first began to be affected, which condition continued to increase in gravity until the date above given. The pain arising from the tooth was followed in a few days by gumboils, which soon opened; and after which the pain in these parts ceased.

Such in brief is the history of this interesting case.

It is evident there was periosteal inflammation, involving the margin and a portion of the floor of the orbit, formed by the maxillary, extending to the canine fossa and alveolar process; with, probably, necrosis of the latter.

The offending tooth was extracted. Its palatine fang was short and thin; the outer larger but also thin. A free opening was made between the cheek and canine fossa, extending three-fourths of an inch upward from the margin of the alveolus. The probe detected a slight necrosis of that process only. The bicuspid could readily be felt in its proper position.

Pressure by means of picked lint, and a bandage around the head was so applied to the contents of the orbit as to gently force the eye downward and backward. One grain of muriate of ammonia was ordered every hour, and morphine at night, sufficient to procure sleep.

The pain in the orbit was, to a great extent, relieved by the pressure, and on the fifth day, the condition of these parts had so much improved that it was discontinued. On the tenth day the muriate of ammonia was also dropped.

The patient improved daily, except a certain degree of tenderness near the infraorbital foramen, which eventually became the seat of an abscess. It was opened Jan. 5th, since which this complication has gradually disappeared.

March 3d. Vision excellent, accommodation perfect, movement of globe good, except downwards, which is gradually improving. A certain amount of periosteal inflammation of the floor of the orbit still remains, but this continues to abate.

The above case fully illustrates the necessity of giving proper attention to certain affections of the teeth, as a means of avoiding, if not in all cases as serious as this one, many, and often important, affections of the eye.

The number of diseases of the eye, arising from "*dental causes*," whose name is legion, is unquestionably much greater than many oculists or dentists are aware of.

Let us hope this subject may receive the earnest attention of all true laborers in dental and ophthalmic surgery.

My acknowledgements are due W. W. ALLPORT, D.D.S., for counsel in the above case.

*Chicago*, *March 8th*, 1866.

